# Implementation experience during an eighteen month intervention to improve paediatric and newborn care in Kenyan district hospitals

**DOI:** 10.1186/1748-5908-4-45

**Published:** 2009-07-23

**Authors:** Jacinta Nzinga, Stephen Ntoburi, John Wagai, Patrick Mbindyo, Lairumbi Mbaabu, Santau Migiro, Annah Wamae, Grace Irimu, Mike English

**Affiliations:** 1 KEMRI Centre for Geographic Medicine Research – Coast, and Wellcome Trust Research Programme, P.O. Box 43640, Nairobi, Kenya; 2 Department of Paediatrics, University of Oxford, Oxford, UK; 3 Division of Child Health, Ministry of Health, Nairobi, Kenya; 4 Department of Paediatrics, College of Health Sciences, University of Nairobi, Nairobi, Kenya

## Abstract

**Background:**

We have conducted an intervention study aiming to improve hospital care for children and newborns in Kenya. In judging whether an intervention achieves its aims, an understanding of how it is delivered is essential. Here, we describe how the implementation team delivered the intervention over 18 months and provide some insight into how health workers, the primary targets of the intervention, received it.

**Methods:**

We used two approaches. First, a description of the intervention is based on an analysis of records of training, supervisory and feedback visits to hospitals, and brief logs of key topics discussed during telephone calls with local hospital facilitators. Record keeping was established at the start of the study for this purpose with analyses conducted at the end of the intervention period. Second, we planned a qualitative study nested within the intervention project and used in-depth interviews and small group discussions to explore health worker and facilitators' perceptions of implementation. After thematic analysis of all interview data, findings were presented, discussed, and revised with the help of hospital facilitators.

**Results:**

Four hospitals received the full intervention including guidelines, training and two to three monthly support supervision and six monthly performance feedback visits. Supervisor visits, as well as providing an opportunity for interaction with administrators, health workers, and facilitators, were often used for impromptu, limited refresher training or orientation of new staff. The personal links that evolved with senior staff seemed to encourage local commitment to the aims of the intervention. Feedback seemed best provided as open meetings and discussions with administrators and staff. Supervision, although sometimes perceived as fault finding, helped local facilitators become the focal point of much activity including key roles in liaison, local monitoring and feedback, problem solving, and orientation of new staff to guidelines. In four control hospitals receiving a minimal intervention, local supervision and leadership to implement new guidelines, despite their official introduction, were largely absent.

**Conclusion:**

The actual content of an intervention and how it is implemented and received may be critical determinants of whether it achieves its aims. We have carefully described our intervention approach to facilitate appraisal of the quantitative results of the intervention's effect on quality of care. Our findings suggest ongoing training, external supportive supervision, open feedback, and local facilitation may be valuable additions to more typical in-service training approaches, and may be feasible.

## Introduction

We have undertaken an intervention study to evaluate whether a multifaceted intervention aimed at implementing evidence based clinical practice guidelines (CPGs) and improving the quality of care works in Kenyan hospitals. The study included eight Kenyan district hospitals from four of the country's eight provinces selected to be broadly representative of this facility type. Within the full intervention package (four hospitals) we aimed to deliver training, guidelines, external supervision, and feedback on progress made in improving care in line with the standards and guidelines provided. We also planned to initiate and support local facilitation to promote implementation. A parallel control group of four hospitals received a minimal intervention. Here we report how the intervention was actually delivered by the implementing team over the 18 months period to answer the question 'what was the intervention'? We also report the views of the hospital health workers to help answer the question 'how well was the intervention delivered'? In separate reports, we have described the development of the guidelines and training [1], a description of the Kenyan health sector more generally, and possible key events at national and hospital levels that might influence responses to the intervention and structure, process, and outcome characteristics characterizing hospitals' quality of care prior to intervention [2]. Measuring whether the intervention results in changes in structure and process aspects of the provision of care for children and newborns will be based on the findings of six-month surveys that assess predominantly structural and process aspects of care. Interpreting these results and considering their generalisability should, however, take into consideration how well the intervention was delivered, and whether it was locally acceptable that are described here.

## Methods

Descriptions of the implementing team's delivery of training, supervision, and feedback are based on prospectively designed and collected records maintained to meet these objectives. These records included research team activity logs and a standardized recording form for documenting, briefly, the main topics of telephone contact with hospitals and facilitators. All such records were reviewed by one author (ME) at the end of the 18-month intervention period, and the nature, timing, and content of interactions with the hospitals were abstracted. In the case of telephone logs, the focus was on identifying the common themes of conversation topics only; a detailed content analysis was not undertaken. Preliminary summaries and interpretations of these data were supplemented and revised using personal reflections of the research team referring to their prospectively collected field notes. The described roles of the facilitators and how these evolved were based on review of the telephone logs, informal discussions during hospital visits, and specific small-group discussions with the facilitators conducted during and at the end of the 18-month intervention project.

To explore how supervision and feedback provided by the implementing team to hospitals and facilitation provided within hospitals were perceived by hospital health workers in the study, and how these aspects of the intervention might have affected its success, we used qualitative research methods now outlined.

### Study Population

Health workers involved in this aspect of the study were selected from all eight hospitals based on the following criteria:

1. Health worker type – medical specialist, medical officer (MO, trained for five to six years with two to eight per hospital), clinical officer (CO, trained for three years with 12 to 20 per hospital), MO intern, CO intern, and nurses (trained for three years with 120 to 250 per hospital).

2. Health workers directly involved in pediatric care at the time of the visit working in the pediatric ward, the maternity unit, the out-patient department (OPD) and the maternal and child health department (MCH).

3. Administrative staff involved in implementation of new policies such as the hospital's medical superintendent, senior nurse, district clinical officer (DCO), health administrative officer (HAO), and those in charge of the various pediatric departments.

4. The hospital selected local facilitators (their selection, background, and roles are fully described in a subsequent section).

### Sampling Procedure

We used a multi-stage sampling procedure. Initially, health workers in hospitals whose duties involved working in or management of the pediatric areas at the time the investigator (JN) visited were considered eligible. Within this sample, health workers of the cadres listed above were purposefully selected with the aim of exploring a wide range of opinions in intervention and control hospitals until the point of saturation in both (when little new was being offered by new interviewees). Data were collected in March and April 2008 from a total of 84 hospital staff (51 in-depth interviews), including administrators, doctors, COs, and nurses (Table 1) approximately 18 months after the start of implementation in the four intervention and four control hospitals.

**Table 1:** Numbers of hospital staff interviewed

	**Intervention**				**Control**				**Tools used**	
**Hospital**	**H1**	**H2**	**H3**	**H4**	**H5**	**H6**	**H7**	**H8**	**Group Interview**	**In-depth Interview**
**Medical Officers**	1	2	1	4	1	1	2	0	0	12
**Clinical Officers**	3	2	4	2	4	2	1	2	2	12
**Medical Officers interns**	0	0	0	0	0	0	3	0	1	0
**Clinical Officers interns**	2	0	0	0	1	0	2	0	2	1
**Nurses**	1	2	3	9	1	3	5	4	6	12
**Administrative Staff**	2	1	1	2	2	1	1	2	1	10
**Hospital Facilitators**	1	1	1	1	0	0	0	0	1	4
**TOTAL**	10	8	10	18	9	7	14	8	13	51
**Total Number of Health Workers studied**										**84**

### Tools for data collection

We reviewed literature describing and defining different aspects of supervision and feedback and aspects of the intervention we thought would be important for promoting improvements in the quality of paediatric care during the sustained intervention [3-8]. Based on these reports and earlier experience exploring the barriers to guideline use in the same hospitals, we developed a semi-structured interview guide to explore health workers' perceptions of the different forms of feedback provided, their experience of supervision provided by the implementing team, and their experience and views on the role and value of the facilitator present in intervention hospitals. This interview guide was pre-tested in the Kenyatta National Hospital, a non-study hospital, and responses analyzed and questions revised prior to use in study hospitals. Where appropriate, additional questions and themes were explored as different issues emerged. All the interviews were conducted in English, each lasting between 20 to 50 minutes. In-depth interviews and small group interviews consisting of two to four persons were conducted. Additional data sources included informal discussions and field diaries of observations and informal discussions kept by one researcher (JN) during visits to hospitals.

### Data Analysis

All the interviews, group discussions, and field notes were transcribed and cleaned by a single researcher (JN). These data were separately coded into themes emerging from the data that either helped us understand how the intervention recipients experienced the process of supervision, feedback, or facilitation or that represented either positive or negative perceptions of these processes. Themes were explored and discussed with other researchers before arriving at an agreed set of simple descriptive codes for analysis using NVivo 7 software (QSR International Pty Ltd 1999–2006). Insights were discussed with all the four facilitators at a meeting with one researcher (JN). During and after this presentation, each of the facilitators gave their accounts of and comments on the research team's interpretation of health worker views from their perspective as a staff member in an intervention hospital. While the main aim was exploration and description of supervision and feedback in intervention hospitals, data from control hospitals were used primarily in a counter-factual sense to determine whether views expressed could be related to the intervention.

## Results

### Part one: delivering the intervention

#### Initial training

Identified hospitals were randomly allocated [1] to two groups of four hospitals at the start of the study. Identical baseline surveys evaluating hospital care within the classical Donabedian framework of structure, process, and outcome [9] were then conducted between 9 July and 19 August 2006 [2]. During these baseline surveys, training was arranged with the administrators of both intervention and control hospitals. We have previously described in detail the training (ETAT+) provided to intervention hospitals [10]. In brief, however, a five and one-half day course was provided incorporating one and one-half days of lecture material combined with three days of small-group, interactive, practical sessions based largely on clinical scenarios and including skills training provided by at least four trained facilitators/instructors. The course also included reflective exercises – a walkabout review of current practice and audit – and end of course, individual testing of participants. Use of standard paediatric admission records (PARs) and CPGs was an integral part of this practical training. We were able train 32 staff from each hospital, of all cadres, hoping to work with the hospital to concentrate on those staff providing services where sick children or newborns are commonly encountered (see Table 2).

**Table 2:** Summary of training provided to study hospitals at the start of the intervention and, for intervention hospitals, during the 18 months intervention period.

	H1	H2	H3	H4	H5	H6	H7	H8
Length of Initial Training (days)	5.5	5.5	5.5	5.5	1.5	1.5	1.5	1.5
Total staff at initial training	33	31	35	29	37	35	43	42
Doctors at initial training	1	2	3	4	1	0	4	1
Clinical Officers at initial training	8	8	11	2	11	2	4	4
Nurses at initial training	24	20	19	23	24	26	25	32
First external follow-up training*								
Length (hours)	6	2	10	4	Control sites were given no further training			
Total Trained	11	9	14	6				
Second external follow-up training*								
Length (hours)	3	2	3	10				
Total Trained	7	14	8	8				
Third external follow-up training*								
Length (hours)	4	10	3	12				
Total Trained	10	24	14	33				
Fourth external follow-up training*								
Length (hours)			3					
Total Trained			27					

For the timing of training see Table 2.

*External follow-up training was provided by the external supervisor, within or near the hospital, at the time of supervisory or survey visits and covered topics mostly but not exclusively related to the original ETAT+ training. Its aim was often to orient staff who had not attended the initial training to the practice guidelines and paediatric admission record forms.

In control hospitals, only the lectures were provided in the form of a one and one-half day seminar aimed at an audience of 40 to 45 health workers providing paediatric services in the hospital. After the training in both intervention and control sites, hospitals were given copies of the Ministry of Health's CPG booklet , copies of wall charts containing the same material, and four copies of three basic reference texts [11-13] for paediatric areas in the hospital. At the conclusion of the training seminar, a 60-minute presentation and discussion of the results of the baseline survey were given, and detailed, printed reports of the survey findings were provided to each senior administrator and department head. The hospitals' administration, all seminar participants, and all staff providing data during the baseline survey were aware that follow-up surveys were planned approximately every six months for 18 months. All training was conducted between 16 September and 2 November 2006, with participation summarized in Table 2.

#### Ongoing training using elements of the same ETAT+ materials

In addition to the initial training, the implementing team (ME, GI and SN) provided intermittent training while conducting supervisory visits (Tables 2 and 3). These were largely conducted as forms of continuous medical education (CME) aimed, if possible, at times when clinical interns rotated. These very occasionally took the form of short local seminars lasting a maximum of one and one-half days and requiring at most two trained instructors. However, in most instances ongoing training was conducted in sessions lasting one to three hours. Within hospitals, staff were also encouraged to organize, by themselves, ongoing CME sessions of approximately 30 to 60 minutes using original ETAT+ training materials given to the hospital at the end of the course.

**Table 3:** Summary of major activities undertaken by the supervisory team with time measured in weeks from the onset of the first intervention hospital training. Control site surveys were undertaken in parallel with those illustrated for the intervention sites

Weeks from onset of intervention	H1	H2	H3	H4
	
1			Baseline training	
2	Baseline training			
4		Baseline training		
6				Baseline training
8		Supervision and feedback		
12 to 13	Supervision and feedback		Supervision and feedback	Supervision and feedback and first follow-up training
	
22 to 26	Survey two	Survey two	Survey two	Survey two
	
	Supervision and first follow-up training	Supervision and first follow-up training	Supervision and first follow-up training	Supervision and second follow-up training
33	Workshop with 4 participants from each hospital to provide feedback to the ministry of health and others on the intervention			
34 to 37	Supervision and feedback	Supervision and feedback	Supervision and feedback	Supervision and feedback
44	Supervision and second follow-up training		Supervision and second follow-up training	
	
48 to 51	Survey three	Survey three	Survey three	Survey three
	
		Supervision and second follow-up training		
55 to 56	Supervision and feedback	Supervision and feedback and third follow-up training	Supervision and feedback and third follow-up training	
61				Supervision and third follow-up training
64				Supervision and feedback
75	Supervision and third follow-up training		Supervision and 4^th ^follow-up training	
	
80 – 84	Survey four	Survey four	Survey four	Survey four

#### Supervision and feedback

Each intervention hospital was linked to lead researchers (H1 and H3, SN and ME: H2 and H4, GI and ME). The aim was for these researchers to try and play a role approximating that of a regional supervisor tasked with implementing government guidelines and improving paediatric hospital care (for timing of these visits, see Table 3). Control hospitals did not receive this supervision and only received written feedback after surveys. As well as the ongoing training aspects outlined above, this role relied on two to three monthly personal visits and involved:

#### Intermittent face-to-face discussions with the hospital administration

These focused on the progress in implementation of guidelines and improving care and local strategies for solving problems in the provision of effective care. These aspects were particularly addressed when providing feedback that often involved a small group discussion with senior hospital staff during the survey to promote immediate problem solving; this was followed six to eight weeks later by a more formal presentation, open to a wider group of senior and other hospital staff, at which written reports (n = 20) were distributed within the site.

An intermittent but visible presence in the hospital demonstrated that an interest was being taken in the hospital's progress. This involved personal visits to each department, informal discussions with staff members on duty, bedside clinical case discussions where the use of the guidelines could be promoted, and observation and discussion of practice and organization of care.

#### Facilitation

At the start of the project, the hospitals were asked to select from among their own staff a facilitator who was either a nurse (three hospitals) or a CO (one hospital). To ensure that this person was available, the hospitals were supported to release their nominee from full-time duties in return for 18 months of locum funding to cover their routine duties. As part of their preparation, the facilitators received three days of training, together with the research team, aimed at building their skills in: characterizing and defining problems; defining barriers to good practice; achievable goal setting; communication skills; negotiation skills; building partnerships; and managing groups and small meetings. Facilitators also received ETAT+ training outside their hospital before the start of the intervention and a second time with their hospital colleagues so that they were completely familiar with the guidelines and job aides, and able to provide support to hospital staff who had not received formal training. To support the facilitator, one of the supervisors (GI, ME and SN) contacted the facilitator every one to two weeks by telephone to provide encouragement and advice and help identify goals, priorities, and strategies for their work. The facilitators received no financial incentives and remained Ministry of Health employees. The major roles undertaken by facilitators, identified from the major themes in telephone follow-up logs, were remarkably consistent across the four intervention sites and are outlined in Appendix 1.

### Part two: Health workers' perceptions on the nature of feedback and supervision provided during the intervention

#### Preferences for and response to feedback

In total, 84 health workers across the eight hospitals contributed data (see Table 1). A number of mechanisms for providing feedback were tried over 18 months in the intervention hospitals by the implementation team. It appeared that staff preferred, in order: power point presentations to an open meeting for all staff; feedback incorporated into CME; written reports; summary sheets; and finally, local performance charts. Power point presentations and CME were favored, according to the health workers, because they were more interactive, less personalized, and provided a forum where all types of health worker and all the pediatric departments met. Additionally, these interactive sessions, which included the hospital administration, increased their involvement in guideline implementation. Written reports were said only to be available to the senior staff of the hospital, and although summary sheets and performance 'run' charts produced by the facilitator were available in all pediatric departments, these were reported to raise little interest among staff, some of whom also found interpreting them difficult:

'I think it [feedback] is good because when you present to people as a multivariate group of people, you do not present to individuals, it's the hospital. So it's not personalized, I think it's a good way of showing us the weaknesses, the good points because we are a mixed lot. Now if you were giving an individualized thing, someone would feel really intimidated (laughs).'

'The performance charts on the walls done by [Facilitator] are a good way of presenting information but I wonder whether everybody in our ward know what they are reflecting, or what they mean, there is a day I tried studying one but ... and [Facilitator] does these charts in the Paeds ward, the MCH, and the OPD, and he does it so well, and when they come out he replaces them, but you find that us, the people he puts them up for, never read them.'

There was a general consensus that the feedback information was accurate, with health workers describing the first feedback after the baseline survey as the only predominantly negative feedback delivered by the study team. There was a subtle preference for receiving feedback from the external study team rather than the local hospital staff or the facilitator, with reports of better turnout and greater credibility with the study team, although some doubted that feedback would achieve anything:

'At first when they came [study team feedback], the figures were a bit low and we were demotivated that we were not doing well, and we knew we had to work and improve things and we gained so much from the training to improve things.'

' [Feedback is] very good and very eye opening. Actually, these feedbacks have helped us identify gaps which without KEMRI [Kenya Medical Research Institute] we would not have been able to identify. So we have been using this feedback and I hope we will continue to use them to address positively these gaps that have been identified and continue to work with the KEMRI team.'

Q: 'Do you think the feedback that KEMRI has been given here has had any impact on the health workers here?'

A: 'I tend to think that it is halfway known. They take very little interest and they tend to think that these are things concerning the administration and [the facilitator] will implement after all, so what is commented on that feedback, very few will come back to check what went wrong – very few.'

Recognition and encouragement of good performance were reported during feedback meetings to be most critical to the health worker, as well as associated improvements in provision of resources and equipment by the hospital administration. Thus, health workers positively associated feedback information with improved pediatric practice attributed to improved motivation to do the correct thing, the provision of reminders, and increasing positive outcome expectancy. Interestingly, in one intervention hospital, locally generated feedback on progress was incorporated into regular hospital management team meetings, and in another initiated in-house client exit surveys:

'It [feedback]' has been very much useful ... when they come and then they check the emergency tray, and then maybe there are some drugs missing like let's say Phenobarb [a drug used for treating convulsions], they will then push the pharmacy to buy the drug because they have come for the supervisory visit. So, the administration will be told that you have such and such drugs missing because you know you may be missing something and you are not aware. Like we were missing a sucker in MCH the last time they came and they brought it up in the feedback then we chased for one and we got it. So these visits are really useful, because they push the administration to provide things that are not there, and we are very happy.'

#### Experience of supervision

Health workers' descriptions of their experience of supportive supervision from the study team could be characterized as guided, experiential learning with provision of open, evaluative information on how to improve care provided to children through the use of guidelines. However, the impact of supervision and feedback was felt to be strongly dependent on individual health workers' appetite for and willingness to change. Direct clinical supervision of patient care by the study team was received with mixed feelings, however, with interns and new staff welcoming the learning opportunity while some health workers felt that the team came to scrutinize mistakes. Interestingly, health workers preferred the study team to help perform some of their clinical duties as a show of support and a better acknowledgement of their responsibilities:

'They were just giving what they found on the ground, and as I said, they were supportive and facilitative, they give the feedback the way they found on the ground and support the team. Where the team was doing well, they would praise them and encourage them on the parts that were missing, and where things were done poorly, they were brain-storming together with the team. They would find out why such a thing was happening and what action should be taken, and normally it was the team that was suggesting how to solve the problem, they were never telling the team what to do, they would just suggest what to do, so they were like counselors.'

'I don't know .... if in your [supervisory] team you have nurse and doctors, then they should be coming and working with us, not just ... so that they know how we are doing. If there is a nurse, let her come with us, we do that midwifery, we deliver, we resuscitate that baby, we see how it goes. But the way you come, it's like looking for mistakes ... to be in our shoes, to know how things are. ... But if you helped, we will not feel like you were wasting our time, but that you were with us and then may be in the end you can even make ... you will have seen how I was working. Like yesterday I heard the doctor saying 'they are always coming here, wasting our time' yet he is busy wanting to do something.'

In control hospitals, health workers continued to report the lack of local supervision and feedback well over a year after the implementation of the guidelines. Where hospital supervision was reported in control hospitals or intervention hospitals prior to the intervention it was characterized as infrequent, haphazard, and in the form of vague departmental visits by the senior staff and the department in-charges. There was no real attempt at internal performance evaluation and feedback.

#### Health workers' perceptions about the role and practice of local facilitation in intervention hospitals

Generally, health workers regarded the facilitators positively and their observations of the facilitator's role were closely associated with those identified by the implementing team (Appendix 1).

(Facilitator): 'my roles are like ... drawing those graphs, giving them feedback reports, CMEs, helping them with some procedures, like doing intra-osseous, then when there are no resources, colluding with the office, the stores, the pharmacist, then see what to do like negotiating with them to do the purchasing.'

The facilitators managed to be guiding and supportive without provoking negative emotions amongst colleagues in all but a few situations that were slowly resolved. Health workers described facilitators as role models, peer educators, a reminder to use the guidelines, in some cases as friendly supervisors and as a link between the health workers and the hospital administration:

'Hey, he [facilitator] is very helpful. You know, he is a link between us and the administration in case there are shortages in terms of supplies; he makes sure we get them or any other problems we are facing. Again, he is always there on the forefront sensitizing people when it comes to ETAT even when you see that people are not willing, and then he is also there to arrange for CME's.'

' [Facilitator] is ... a tank of support and he ... was my conscience when I was working in pediatrics ... because may be there were times when I would be tired ..., maybe I [had] just finished a ward round and I just want to run away ... but then he would remind me.'

However, some clinicians expressed their dissatisfaction that a nurse as a facilitator might influence clinical management decisions, illustrating the somewhat rigid thinking about the hierarchy of roles seen in Kenyan hospital care. Interestingly, although they were regarded as leaders in the implementation of the programme, there was also a prevalent perception that their main work was as data collectors for the study team. Linked to this there was a misplaced perception that the four facilitators must have been receiving a financial incentive that explained their enthusiasm for their role.

'Well, I guess he's actually doing what he ... what he's supposed to do or what he can actually do within his jurisdiction, but I think it would have been more effective if it was a clinician rather than a nursing staff ... you get ... so that you're part and parcel of the ward round and you're part and parcel of making the decisions...'

(Facilitator): '...in fact there is someone who was saying, ' [facilitator] is getting 60,000 from KEMRI per month, on top of his salary, wacha akuje afanye kazi (let him come and work).' Imagine that situation where people do not even want to see you.'

The facilitators, in describing their experience in the implementation of guidelines, characterized it as: emotionally taxing, hectic, and requiring considerable patience and persistence both with the administration and the staff:

(Facilitator): 'But at the same time, its hectic, there is a lot of headache as a facilitator. At times, you might tell someone that this one is supposed to be done this way, then you find that person repeating the same mistake you corrected, you have to swallow your anger and start afresh. So, that process of training and reminding people on the same things everyday, and at times some people are just slow, you just have to adjust and accept them the way they are. So at times you want to get annoyed but you have to cover that annoyance and you don't want to show anyone that you are annoyed, sometimes you wonder whether may be you are the one who is not handling them the right way.'

The most challenging experiences, the facilitators reported, were in the OPD that predominantly serves adults while providing services to sick children at nights and weekends, and with the COs. These departments and individuals were reported to embrace change the least well while the pediatric wards were felt to have shown the best improvement.

(Facilitator): 'For me, I think people believe that children should be seen separately from the adults so the children landing in OPD during odd hours are not getting the proper care, it's just negligence, because sometimes a clinician will say, 'me, I don't want to see children'.'

Success stories described by the facilitators that illustrate their role to promote change included: having enabled networking within hospitals; developing a role as team builders and team players; building collaborative relationships with the administration; and, more importantly, a sense that they were contributing to a reduction in child mortality and morbidity in their hospitals.

(Facilitator): '(sighs) it has come with a lot of things. One thing, it has taught me how to network with people, that one is for sure. This programme has made me be a team builder. Before, I just used to make sure that everything that I do, I do it right; but when I became a facilitator, it dawned on me that I have to make the other person do it perfectly. So it has made me be a team player to ensure that other people do it right. So I came from being an individual to interacting with the other people to talking to the clinicians, talking to the other nurses, getting very close to the administration especially, getting things done.'

Among all the facilitators, there was a general consensus that facilitation will have to be maintained permanently for sustainable implementation in the different hospitals.

(Facilitator): 'Sustainability really depends on who is on the ground. I think, as for me it is still my responsibility to maintain ETAT.'

## Discussion

It is becoming increasingly apparent that hospital care for children is poor in many low-income settings [14-16]. While there are proposed tools and international calls to change this situation [17,18], there have been only a handful of studies attempting to evaluate and understand how to change such hospitals [19]. More broadly, we still know little about how to change health worker behavior and improve their performance in low-income settings [20]. We have therefore attempted to summarise the actual delivery of training, supervision, feedback, and facilitation provided during an 18-month intervention project aimed at improving paediatric and newborn care in Kenyan district hospitals. Understanding the 'nuts and bolts' of the process of intervention is essential when attempting to draw inference about its degree of success and guide the development of improved strategies in the future. While the team describing the intervention and supplying the intervention are largely the same, potentially introducing bias in such a narrative approach, we attempted to limit this by establishing prospective data collection and revising our qualitative findings after review and discussion with hospital staff. Training was clearly a key component of the intervention, and in particular the ability to offer follow-up, less formal training in the intervention hospitals varying from 30 to 60 minutes locally arranged CME meetings to a few one and one-half day seminars conducted by external supervisors (see Tables 2 and 3) may be key. Such ongoing training was felt to be important to address problems of staff turnover and initial non-attendance. Importantly, this ongoing training or orientation need was also addressed by on-the-job support and advocacy provided by the facilitator and key allies. The need for ongoing training makes it easy to see why one-off episodes of in-service training, a very commonly used intervention, may fail. For example, in the largest control hospital, other than the paediatrician, no member of the ward-based clinical team present at 18 months had attended the introductory seminar. In-service seminars, unless they are linked to clear and long-term staff deployment plans, therefore seem an extremely poor way to institutionalize new practices in most hospitals.

In all four control hospitals, the relationship between the hospital management and the research team remained formal and distant, representing, we feel, a fairly typical scenario when implementing new practices in the public sector. In contrast, in the intervention hospitals the implementing team was able to build relationships with the hospitals. Such local leadership is felt to be critical to achieving change [21]. A variety of actors assumed leadership roles in collaboration with the implementing team in attempting to improve care in intervention hospitals. At two sites, the facilitator assumed much of the leadership role supported by individually active ward or outpatient based staff who had also been trained. This devolved leadership role was possible because the medical superintendents provided visible endorsement for attempts to improve care although restricting their personal roles largely to authorizing activities, solving administrative or resource problems where possible, and making expectations of progress clear. At another site, the medical superintendent (also a paediatrician) was strongly supportive of the facilitator. At the fourth intervention site, the facilitator and key allies were supported by a senior management role primarily adopted by the administrative officer and two of the senior nurses. One result of the intervention approach was, therefore, the establishment of a largely informal but nonetheless identifiable leadership grouping in each intervention site that was not apparent in the control sites. Such groupings provided both support to the facilitator and a key constituency with which the research team could communicate with the hospital more broadly. Interestingly, these groupings remained remarkably stable over the 18 months of the intervention.

The research team, in its external support supervision role, tried to be sensitive to the fact that overcritical feedback might be damaging. In general, therefore, we attempted to combine positive messages about progress being made – and encouraging further progress – with feedback on areas where little or no progress was being made. Health workers found the supervision generally supportive and the feedback credible, and both may be important in promoting change [22,6]. They also expressed a clear preference for group feedback that included hospital administrators where there were opportunities for discussion, problem solving, and goal setting. Although attempts at 'benchmarking' with other intervention sites promoted discussion, this approach and performance 'run-charts' were not highly regarded in these relatively large and complex organisations.

From the perspective of the research team, the feedback provided and the discussions these prompted appeared open and not at all defensive. However, while an obvious solution often was easily identified and actors nominated, the ability to deliver local solutions was sometimes limited. For example, hospitals might simply not find a local supplier of missing resources even though they were prepared to use local funds to purchase them. On other occasions the ability to address problems was affected by under-staffing, particularly for nurses, and it was therefore not that uncommon for a problem to be a recurring issue. A more particular challenge facing the facilitators was explicit or implicit refusal of a minority of health workers to change, although the majority of staff seemed to find that the facilitators supported, motivated, and sometimes inspired them, making them as potentially valuable as agents for change as formal leaders [23].

## Conclusion

What health workers probably require from administrators or supervisors is leadership that is 'transformational, requiring leaders to be able to empower and motivate them, define and articulate a vision, build and foster trust and relationships, adhere to accepted values and standards, and promote acceptance of change [8]'. We believe the combination of external supervision, local administrative support, feedback, and specific facilitation helped in part to achieve this within existing resource constraints in the intervention hospitals. In contrast, in control hospitals local attempts at improvement seemed less common and more haphazard. Although such an intervention programme requires considerable initial investment, two to three days supervision every two to three months for hospitals may be feasible more widely. Furthermore, in our setting, where many nurses are unemployed, the cost of a facilitator for one year is less than $5,000, comparing very favourably with the cost of a single, full Integrated Management of Childhood Illnesses (IMCI) training for 30 health workers of approximately $20,000. The sustained intervention package we have carefully described, if proven to change practice, may therefore provide a workable model for wider efforts at improving hospital care for children and newborns.

## Competing interests

The authors declare that they have no competing interests.

## Authors' contributions

The idea for the study was conceived by ME who obtained the funding for this project. Preparation for and conduct of the study was undertaken by all authors. JN undertook all the interviews and the qualitative analysis with support from PM, LM, and ME. ME reviewed data and summarized the implementation team's process of intervention. ME and JN produced the draft manuscript to which all authors contributed during its development. All authors approved the final version of the report.

## Appendix 1

### Facilitators' major activities

Promoting the uptake and completion of the Paediatric Admission Record Form, including frequency of use and degree of completeness. This involved local audit, group and individual feedback, and one-on-one coaching that on occasion required delicate handling of those resistant to this new practice.

Organising, advertising, and providing short hospital CME sessions on the CPGs, including attempts to target those who had not attended initial training and those resistant to adopting new practices.

Distributing copies of CPG booklets and providing one-on-one orientation on the CPGs through bedside coaching for new staff rotating into the paediatric areas.

Liaising with hospital's clinical departments, stores, pharmacy, kitchen, and administration to tackle organizational or resource issues. In most cases, attempts to establish a 'core quality team' were not successful because of the difficulty in arranging or executing meetings. Thus 'virtual' core groups were formed with the facilitator becoming the channel for communication to permit consensus decisions on priorities for action and mechanisms for action.

Liaison with clinical and nursing staff through ward and other meetings to reorganize patient flow where possible, and to promote hand-washing and appropriate patient monitoring, including the use of feeding/monitoring charts.

Production and distribution of 'run-charts' demonstrating progress in such issues as: proportion of admitted children in whom a PAR was used; proportion of malaria cases with a fully documented clinical assessment; and proportion of dehydration cases with an appropriate fluid prescription.

Introduction of mortality or case-based audit to identify areas of care requiring improvement
